# Supplementary material for: Occurrence of Chlamydiaceae and Chlamydia felis pmp9 Typing in Conjunctival and Rectal Samples of Swiss Stray and Pet Cats
Source: Pathogens. 2021 Jul 28;10(8):951. doi: 10.3390/pathogens10080951 (PMC8400119; doi:10.3390/pathogens10080951)
Supplement: Supplementary file 1 [file pathogens-10-00951-s001.zip › pathogens-1283525-supplementary.pdf]

pathogens

MDPI

Supplementary Table S1. Individual and mean Ct (μ Ct) values of sample duplicates obtained with flocked swabs (1) or cytology brushes (2) and differences of μ Ct values within $n = 35$ sample pairs (Δ μ Ct = μ Ct_2 - μ Ct_1).

Nr	Cat	Flocked Swabs				Cytolog Brushes				Δ μ Ct
		Eye	Ct of Duplicates		μ Ct_1	Eye	Ct of Duplicates		μ Ct_2	
1	KE1	Left	26.1	26.0	26.1	Right	27.1	27.3	27.2	1.1
2	KE2	Left	23.9	24.0	24.0	Right	27.5	27.5	27.5	3.5
3	KN3	Left	24.4	24.3	24.4	Right	27.4	27.5	27.5	3.1
4	KU1	Left	23.8	23.9	23.9	Right	26.4	26.4	26.4	2.5
5	KU6	Right	22.5	22.4	22.5	Left	24.8	24.8	24.8	2.3
6	KU7	Left	27.7	27.7	27.7	Right	30.0	29.8	29.9	2.2
7	KZ2	Left	25.2	25.1	25.2	Right	28.9	29.0	29.0	3.8
8	KZ8	Right	22.3	22.2	22.3	Left	27.6	27.6	27.6	5.3
9	KZ9	Right	24.0	23.8	23.9	Left	26.4	26.4	26.4	2.5
10	KZ10	Left	23.9	23.9	23.9	Right	26.8	26.8	26.8	2.9
11	KZ13	Right	23.5	23.4	23.5	Left	26.0	26.1	26.1	2.6
12	KZ15	Right	22.3	22.4	22.4	Left	26.6	26.7	26.7	4.3
13	LD2	Right	25.3	25.4	25.4	Left	28.6	28.6	28.6	3.2
14	LD3	Left	23.9	23.9	23.9	Right	28.5	28.5	28.5	4.6
15	LE1	Right	26.1	26.2	26.2	Left	27.8	27.8	27.8	1.6
16	LE2	Left	26.6	26.6	26.6	Right	30.1	30.1	30.1	3.5
17	LJ1	Left	29.2	29.1	29.2	Right	29.4	29.4	29.4	0.2
18	LK1	Right	25.2	25.1	25.2	Left	26.6	26.5	26.6	1.4
19	LK2	Left	25.1	25.1	25.1	Right	30.1	30.0	30.1	5.0
20	LK3	Right	26.3	26.3	26.3	Left	28.4	28.6	28.5	2.2
21	LK5	Left	26.1	26.1	26.1	Right	28.3	28.3	28.3	2.2
22	LK6	Right	25.0	24.9	25.0	Left	26.4	26.4	26.4	1.4
23	LO2	Right	25.2	25.2	25.2	Left	27.6	27.7	27.7	2.5
24	LP1	Left	27.6	27.5	27.6	Right	29.8	30.0	29.9	2.3
25	MC1	Left	27.1	27.0	27.1	Right	29.1	29.1	29.1	2.0
26	MD1	Right	28.2	28.0	28.1	Left	26.4	26.4	26.4	-1.7
27	ME3	Left	23.4	23.5	23.5	Right	28.5	28.7	28.6	5.1
28	ME4	Right	23.9	23.9	23.9	Left	25.5	25.6	25.6	1.7
29	MJ1	Right	25.9	25.9	25.9	Left	24.9	24.9	24.9	-1.0
30	MJ2	Right	26.9	26.9	26.9	Left	27.7	27.7	27.7	0.8
31	MJ4	Left	24.4	24.5	24.5	Right	27.8	27.8	27.8	3.3
32	ML4	Left	25.7	25.8	25.8	Right	32.3	31.8	32.1	6.3
33	ML7	Left	27.0	26.9	27.0	Right	30.1	30.2	30.2	3.2
34	ML8	Left	25.9	26.0	26.0	Right	28.8	28.9	28.9	2.9
35	ML10	Right	28.4	28.4	28.4	Left	27.3	27.3	27.3	-1.1

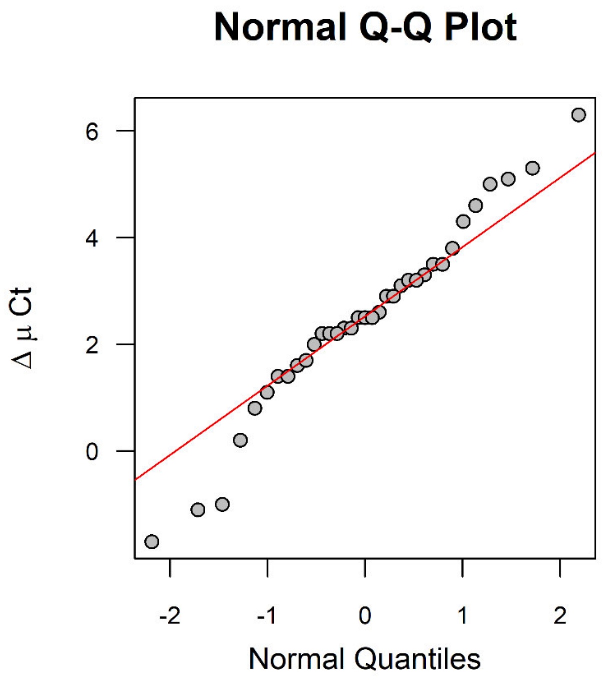

Supplementary Figure S2. Normal Q-Q plot of differences in mean Ct values between flocked swabs and cytology brushes: Differences in mean Ct values between $n = 35$ sample pairs (Δ μ Ct) obtained with flocked swabs (1) and cytology brushes (2) appear to follow a normal distribution. Normal Q-Q plot with $\Delta\ \mu\ Ct = \mu\ Ct_2 - \mu\ Ct_1$ plotted against normal quantiles.

Supplementary Table S3. Results of statistical analysis performed in this study.

Method	Statistical Test	Output	Interpretation
Feline albumin qPCR	Shapiro-Wilk normality test	*p*-value = 0.446	Not significant Normal distribution
Feline albumin qPCR	Paired t-test (two-sided)	*p*-value = 8.768×10^{-10}	Significant
Feline albumin qPCR	Paired t-test power calculation	power = 0.9999995	Sufficient sample size
23S qPCR (comparing symptomatic and asymptomatic cats)	Pearson's Chi-squared test	*p*-value = 8.953×10^{-14}	Significant
23S qPCR (comparing total stray and pet cats)	Pearson's Chi-squared test	*p*-value = 0.155	Not significant
23S qPCR (comparing symptomatic stray and pet cats)	Pearson's Chi-squared test	*p*-value = 4.956×10^{-8}	Significant
23S qPCR (comparing sampling sites)	Pearson's Chi-squared test	*p*-value = 1.805×10^{-8}	Significant

Supplementary Table S4. Positive samples, sampling site, mean Ct values (µ Ct Values) of sample duplicates obtained from the 23S rRNA qPCR, calculated chlamydial genome copy numbers per µl of sample DNA and per swab as well as chlamydial DNA content per swab in ng.

Sample	Sampling Site	*Chlamydia* Species	µ Ct Values	Copies / µl DNA	Copies / Swab	DNA / Swab [ng]
A6E.1e	Eye	*C. felis*	25.0	20,300.00	1,020,000	1.114
A6E.2e	Eye	*C. felis*	24.0	36,600.00	1,830,000	2.006
A7E.1e	Eye	*C. felis*	36.1	2.36	1180	0.001
AO47.2e	Eye	*C. felis*	35.9	9.43	471	0.001
BC2.1e	Eye	*C. felis*	35.1	16.20	812	0.001
BC2.2e	Eye	*C. felis*	34.9	24.50	1220	0.001
BF1.1e	Eye	*C. felis*	31.5	821.00	41,000	0.045
BF1.2e	Eye	*C. felis*	36.1	9.56	478	0.001
BT2e	Eye	*C. felis*	34.4	33.40	1670	0.002
BX2.1e	Eye	*C. felis*	37.2	5.04	252	0.000
BX2.2e	Eye	*C. felis*	32.9	98.30	4910	0.005
BY1.2e	Eye	*C. felis*	33.9	47.90	2400	0.003
BY2.1e	Eye	*C. felis*	31.3	884.00	44,200	0.048
BY2r	Rectum	*C. felis*	33.0	327.00	16,400	0.018
CS2.1e	Eye	*C. felis*	26.0	7740.00	387,000	0.424
CS2.2e	Eye	*C. felis*	32.0	125.00	6270	0.007
CS4	Unknown	*C. felis*	29.8	1,030.00	51,700	0.057
CS5.1e	Eye	*C. felis*	33.7	90.20	4510	0.005
CS5r	Rectum	*Chlamydiaceae*	36.5	7.58	379	0.000
CS6	Unknown	*C. felis*	31.4	384.00	19,200	0.021
CS8.1e	Eye	*C. felis*	33.2	121.00	6040	0.007
CS8.2e	Eye	*C. felis*	29.8	1030.00	51,300	0.056
DD2.2e	Eye	*C. abortus*	36.5	9.31	465	0.001
ED7.1e	Eye	*C. felis*	37.9	5.50	275	0.000
ED7.2e	Eye	*C. felis*	36.4	14.20	711	0.001
ED8.1e	Eye	*C. felis*	32.0	227.00	11,300	0.012
ED8.2e	Eye	*C. felis*	32.8	141.00	7060	0.008
EI1.1e	Eye	*C. felis*	28.5	1670.00	83,700	0.092
EI1.2e	Eye	*C. felis*	29.8	688.00	34,400	0.038
EI2e	Eye	*C. felis*	30.8	352.00	17,600	0.019
EI3.1e	Eye	*C. felis*	27.9	2520.00	126,000	0.138
EI3.2e	Eye	*C. felis*	26.0	9020.00	451,000	0.494
EI3r	Rectum	*C. felis*	28.4	1780.00	89,200	0.098
EI4.1e	Eye	*C. felis*	25.2	16,000.00	800,000	0.876
EI4.2e	Eye	*C. felis*	22.3	112,000.00	5,580,000	6.114
EI4r	Rectum	*C. felis*	32.8	86.10	4,310	0.005
EK1.1e	Eye	*C. felis*	34.2	33.90	1,700	0.002
EK1.2e	Eye	*C. felis*	30.5	792.00	39,600	0.043
EM01e	Eye	*C. felis*	28.8	2100.00	105,000	0.115
EM03.2e	Eye	*C. felis*	25.7	12,200.00	608,000	0.667
EM04e	Eye	*C. felis*	28.6	2390.00	120,000	0.131
EM12.1e	Eye	*C. felis*	31.2	532.00	26,600	0.029
EM12.2e	Eye	*C. felis*	34.0	106.00	5290	0.006
EM13.2e	Eye	*C. felis*	27.2	5140.00	257,000	0.282
ES2.2e	Eye	*C. felis*	31.8	204.00	10,200	0.011
F09r	Rectum	*C. felis*	29.5	591.00	29,500	0.032
F13.1e	Eye	*C. felis*	31.4	125.00	6270	0.007

F13.2e	Eye	*C. felis*	30.5	231.00	11,500	0.013
F18.1e	Eye	*C. felis*	29.9	290.00	14,500	0.016
F18.2e	Eye	*C. felis*	33.3	26.90	1340	0.001
F28.1e	Eye	*C. felis*	32.0	77.00	3850	0.004
F28.2e	Eye	*C. felis*	33.8	22.20	1110	0.001
F33.1e	Eye	*C. felis*	29.8	158.00	7920	0.009
F33.2e	Eye	*C. felis*	30.4	104.00	5210	0.006
F33r	Rectum	*C. felis*	23.6	12,700.00	636,000	0.697
F34.1e	Eye	*C. felis*	29.4	225.00	11,300	0.012
F36.1e	Eye	*C. felis*	25.1	11,000.00	548,000	0.601
F36.2e	Eye	*C. felis*	21.8	97,600.00	4,880,000	5.347
F36r	Rectum	*C. felis*	25.3	10,300.00	517,000	0.567
F37.1e	Eye	*C. felis*	31.8	122.00	6080	0.007
F37.2e	Eye	*C. felis*	32.1	99.90	5000	0.005
F52.2e	Eye	*C. felis*	31.2	90.90	4550	0.005
F52r	Rectum	*Chlamydiaceae*	33.2	22.50	1120	0.001
F53.2e	Eye	*C. felis*	37.7	0.93	46	0.000
KZ06r	Rectum	*C. felis*	26.6	3880.00	194,000	0.213
KZ16.1e	Eye	*C. felis*	29.9	537.00	26,900	0.029
LD1.2e	Eye	*C. abortus*	38.1	0.69	34	0.000
LD4.1e	Eye	*C. abortus*	36.4	1.50	75	0.000
LG1.1e	Eye	*C. felis*	31.7	62.40	3120	0.003
LG1.2e	Eye	*C. felis*	31.1	99.50	4970	0.005
LG1r	Rectum	*C. felis*	37.0	1.46	73	0.000
LG3e	Eye	*C. felis*	29.9	230.00	11,500	0.013
LG5.1e	Eye	*C. felis*	34.3	10.30	517	0.001
LG5.2e	Eye	*C. felis*	37.6	1.00	50	0.000
LG6e	Eye	*C. felis*	28.4	602.00	30,100	0.033
LQ1.1e	Eye	*C. felis*	32.0	39.60	1980	0.002
LQ1.2e	Eye	*C. felis*	31.4	56.60	2830	0.003
LQ2.2e	Eye	*C. felis*	33.3	22.80	1,140	0.001
LQ4.1e	Eye	*C. felis*	26.1	3430.00	171,000	0.188
LQ4.2e	Eye	*C. felis*	25.6	4970.00	248,000	0.272
LQ4r	Rectum	*Chlamydiaceae*	36.3	1.88	94	0.000
LQ5e	Eye	*C. felis*	31.6	75.90	3790	0.004
LQ6.1e	Eye	*C. felis*	31.7	73.50	3680	0.004
LQ6.2e	Eye	*C. felis*	29.9	248.00	12,400	0.014
LR1.2e	Eye	*C. felis*	29.6	304.00	15,200	0.017
LR2.1e	Eye	*C. felis*	27.6	1260.00	62,800	0.069
LR2.2e	Eye	*C. felis*	27.7	1170.00	58,700	0.064
LR2r	Rectum	*C. felis*	24.2	13,100.00	655,000	0.718
LR3e	Eye	*C. felis*	28.3	754.00	37,700	0.041
LR4.1e	Eye	*C. felis*	31.2	98.20	4910	0.005
LR4.2e	Eye	*C. felis*	28.9	500.00	25,000	0.027
LR4r	Rectum	*Chlamydiaceae*	31.4	87.90	4390	0.005
MB9.1e	Eye	*C. felis*	29.8	204.00	10,200	0.011
MB9.2e	Eye	*C. felis*	29.4	270.00	13,500	0.015
MK2.1e	Eye	*C. felis*	35.0	4.75	237	0.000
MK2.2e	Eye	*C. felis*	31.9	41.80	2090	0.002
MK2r	Rectum	*C. felis*	27.4	2460.00	123,000	0.135
MK3e	Eye	*C. felis*	28.4	523.00	26,100	0.029

MK3r	Rectum	*Chlamydiaceae*	27.4	1120.00	55,900	0.060
MM1.1e	Eye	*C. felis*	35.7	3.50	175	0.000
MM2.1e	Eye	*C. felis*	34.8	6.87	344	0.000
MM3.1e	Eye	*C. felis*	33.8	14.20	708	0.001
MM3.2e	Eye	*C. felis*	31.5	73.00	3650	0.004
MM4.1e	Eye	*C. felis*	31.5	74.60	3730	0.004
MM4.2e	Eye	*C. felis*	30.1	218.00	10,900	0.012
MM6e	Eye	*C. felis*	33.0	28.00	1400	0.002
MM7.1e	Eye	*C. felis*	31.1	104.00	5190	0.006
MM7.2e	Eye	*C. felis*	36.6	2.25	113	0.000
MM7r	Rectum	*Chlamydiaceae*	36.2	3.06	153	0.000
MN1.2e	Eye	*C. felis*	32.0	57.80	2890	0.003
MO1.1e	Eye	*C. felis*	30.1	215.00	10,700	0.012
MO1.2e	Eye	*C. felis*	28.6	587.00	29,300	0.032
MO3.1e	Eye	*C. felis*	29.7	286.00	14,300	0.016
MO3.2e	Eye	*C. felis*	30.4	175.00	8770	0.010
MP1e	Eye	*C. felis*	34.8	8.02	401	0.000
MP2.2e	Eye	*C. felis*	28.9	516.00	25,800	0.028

Supplementary Table S5. Number of stray and pet cats and number of samples from different sampling sites (conjunctival, rectal, unknown sampling site and total swabs) per year and canton.

Population	Year	Canton	Cats	Conjunctival Swabs	Rectal Swabs	Unknown Swabs	Total Swabs
**Stray cats**			**309**	**413**	**301**	**2**	**716**
	2017	FR	28	30	28	0	58
		NW	35	50	33	2	85
	2018	FR	29	31	28	0	59
		NW	62	77	61	0	138
	2020	BE	34	50	32	0	82
		NW	57	82	55	0	137
		OW	64	93	64	0	157
**Pet cats**			**86**	**152**	**86**	**0**	**238**
	2020	ZH	77	135	77	0	212
	2021	ZH	9	17	9	0	26
**Total cats**			**395**	**565**	**387**	**2**	**954**
